# Effects of Nordic Walking on Oxidant and Antioxidant Status: Levels of Calcidiol and Proinflammatory Cytokines in Middle-Aged Women

**DOI:** 10.1155/2018/6468234

**Published:** 2018-03-20

**Authors:** Wanda Pilch, Łukasz Tota, Anna Piotrowska, Ewa Śliwicka, Olga Czerwińska-Ledwig, Roxana Zuziak, Łucja Pilaczyńska-Szcześniak

**Affiliations:** ^1^Department of Cosmetology, Faculty of Physiotherapy, University of Physical Education, Cracow, Poland; ^2^Department of Physiology and Biochemistry, Faculty of Physical Education and Sport, University of Physical Education, Cracow, Poland; ^3^Department of Hygiene, Poznań University of Physical Education, Poznań, Poland

## Abstract

**Objectives:**

Nordic walking (NW) is relatively new and popular type of physical exercise with less studied effects than other sports activities. The aim of the study was to analyze possible changes in somatic indices, oxidant and antioxidant status, interleukins, and calcidiol levels in middle-aged women after a 12-week NW training program.

**Study Design:**

In this study, we examined the effects of NW training on selected measures and changes in body weight, fat mass, and calcidiol levels.

**Methods:**

The study group consisted of 13 women (46 ± 4.2 years), who took part in trainings. Before and after the training program, some anthropometric indices were determined and selected biochemical parameters were measured in blood.

**Results:**

NW training led to a significant decrease of the total body mass and fat mass and to an increase in lean body mass (*p* < 0.05). It also contributed to a significant increase in total antioxidative status (TAS) and calcidiol levels (*p* < 0.05). Before training, a reverse correlation between IL-6 and total oxidative capacity (TOC) levels (*p* < 0.05) was found, while after training between IL-6 and calcidiol levels (*p* ≤ 0.001).

**Conclusions:**

12-week NW training undertaken by premenopausal women not only has a positive effect on body composition but also on the plasma antioxidative capacity.

## 1. Introduction

The impact of physical activity on health has been a subject of extensive interest in recent years. There has been a visible rise in the number of people undertaking various forms of physical activity, including Nordic walking. Regular physical exercise, regardless of individuals' age, does contribute to maintaining good health and improving physical fitness and, in particular, in maintaining appropriate body mass [1–3]. Visceral fat functions as an endocrine and paracrine organ secreting a number of adipokines and cytokines that might contribute to the development of metabolic diseases [4, 5]. It is also associated with hepatic and peripheral insulin resistance [6–8].

It is well known that physical activity improves muscle mass, regardless of one's age, and has a positive effect on muscle strength [9]. Skeletal muscles constitute a highly specialized tissue which features excellent plasticity in response to external stimuli, for example, training. Depending on their type, physical exercises induce a number of phenotypic and physiological changes such as mitochondrial biogenesis, muscle fiber transformation, angiogenesis, improvement of insulin sensitivity, and cytoprotection [10]. Physical exercise stimulates the production of reactive oxygen species (ROS), which may have positive or negative effects on health, depending on their level, exposure time, and one's physical fitness. Physically active individuals display a higher level of adaptation and are, to a greater extent, less susceptible to the adverse effects of the ROS. In women, a lower lean body mass results from a reduction of skeletal muscle mass which is visible between 30 and 50 years of age and intensifies in postmenopausal women [11]. According to Fulle et al. [12], reactive oxygen and nitrogen species (RONS) may induce a reduction in muscle mass and strength due to changes in the functioning of the mitochondrial respiratory chain, with aging muscles becoming more susceptible to exercise-induced oxidative stress [13], as evidenced by increased lipid peroxidation, protein oxidation, and DNA damage.

Vitamin D is necessary for maintaining the structural integrity and function of muscles [14]. Wiseman [15] showed that both 1,25-dihydroxycholecalciferol [1,25 (OH)_2_D_3_] and ergocalciferol (vitamin D2) functioned as membrane antioxidants protecting the cell membrane from damage by free radicals.

In view of the above, it remains paramount to choose training loads that would ensure a balance between the production of RONS and maintaining proper muscle mass and good physical fitness [16]. One such training type can be Nordic walking—a fitness walking activity performed with special walking poles. Nordic walking significantly engages the upper body muscles which usually remain passive during regular walking [17].

The present study aimed at an analysis of possible changes in somatic indices, oxidative stress, and inflammatory markers, as well as calcidiol blood levels in middle-aged women after a 12-week Nordic walking training program.

## 2. Methods

### 2.1. Participants

The study sample consisted of 13 women aged between 40 and 50 years (mean ± SD: 46 ± 4.2), and body height was between 158 and 170 cm (mean ± SD: 164.9 ± 5.4). The participants were required to submit a medical confirmation of fitness to perform physical exercise. They were asked not to receive any pharmacological treatment (for hypertension, hyperlipidemia, or hormonal) or take antioxidative and other dietary supplements for at least four weeks prior to the commencement of the study. The women were also required to abstain from alcohol consumption for at least two weeks before and during the trial. All participants were also asked to maintain their regular dietary habits and fill in the International Physical Activity Questionnaire (IPAQ, abridged version) [18] concerning their physical activity. IPAQ is a questionnaire containing questions about the person's activity in the last 7 days. The subject responds to the questions whether and how much time she/he performed in the last week of activity with high and moderate intensity, in addition to how much time is spent daily in sitting and walking. IPAQ questionnaire responses are converted into three categories: 1 means low physical activity; 2, moderate; and 3, high. Women whose level of physical activity was insufficient to achieve at least moderate (2) were qualified for the study.

In accordance with the Declaration of Helsinki, the participants had been informed about the purpose and methods of the study and the possibility to discontinue their participation at any given stage of the trial as well as they gave their written consent to participate. The study was officially approved by the Bioethical Commission of the Regional Physicians' Chamber (approval number 137/KBL/OIL/2013).

### 2.2. Training Program

The Nordic walking training classes lasted 12 weeks, from March to May, and encompassed three 90-minute sessions a week under an instructor's supervision. Each woman took part in 36 training sessions. Before the commencement of the training program, the women had attended three instruction classes on the proper Nordic walking technique. The length of the walking poles was individually adjusted for each participant [17].

Each training session took place in the morning and comprised three parts: warmup (15.0 ± 2.0 min), main part (60.0 ± 4.2 min), and cooldown (15.0 ± 1.2 min). The warmup consisted of stretching exercises and walking exercises with the poles, preparing the body for the main training part which included aerobic, continuous, and interval walking exercise in different terrains. The cooldown phase included relaxation exercises.

The participants kept training diaries, and after each session recorded the length of the covered distance and training intensity assessed with a heart rate monitor (Polar RS400, Finland). The training volume was presented as the covered distance (km) during training.

The selection of exercise intensity assumed maximal mobilization of body fat reserves during regular training. An effective use of energy resources in the form of free fatty acids (FFA) was to be obtained at an exercise intensity of 50 ± 1.2% HRmax in the main part of the first mesocycle, 60 ± 2.3% in the second mesocycle, and 65 ± 3.1% in the third mesocycle.

HRmax was estimated according to the formula: HRmax = 211−0.64 (years) [19], which amounted to 181.9 ± 2.7 bpm for the study group. In the main part of each training session, the women maintained the designated heart rate, which was monitored with a Sport Tester.

According to the data supplied by the Institute of Meteorology and Water Management in Warsaw in 2014, the sum of insolation for the city of Kraków and suburban areas in March was in the range between 110 and 120 hours and in April 140–160 hours and was approximately 10–30 hours higher than the long-term average for years 1971–2000. In May, the total insolation equals to 200–220 hours, which was also 10–30 hours more than the long-term average for this month.

### 2.3. Somatic Measurements

The body composition measurements were taken before and after the training program, in the morning in a fasting state. The women's body composition was assessed using bioelectrical impedance analysis (Jawon Medical, IOI 353, South Korea) and body height (BH) with an anthropometer (Martin, USA). Other measurements included body mass (BM), percent body fat (F%), fat mass (FM), lean body mass (LBM), and body mass index (BMI).

### 2.4. Biochemical Analysis

A day before the commencement of training and a day after its completion, blood samples were drawn from the participants' antecubital vein in accordance with all safety standards. The samples were collected into EDTA blood collection tubes (6 ml BD Vacutainer® EDTA tubes, Franklin Lakes, NJ, U.S.A.) in order to extract blood plasma and separately for blood serum (6 ml BD Vacutainer Serum tubes, Franklin Lakes, NJ, U.S.A.). Blood samples for serum obtainment were collected into tubes containing coagulation activator which after 20 minutes of clotting at room temperature were centrifuged at RCF 1000 ×g for 15 minutes at 4°C (MPW 351R, Poland), and the obtained serum was stored at a temperature of −20°C until the time of determination. All stages of serum preparation were conducted in an acrylic glovebox station for anaerobic work (MBRAUN, MB GB 2202, Germany) with the use of nitrogen (Linde Gaz, Poland) in order to prevent oxidation of sample components. We did not note hemolysis or lipemia in any of the samples. Total antioxidant status (TAC) and total oxidative capacity (TOC) of the plasma were determined with the use of commercial diagnostic kits (DM P-4100, DM P-4200, LDN Labor Diagnostika Nord GmbH & Co., Germany). Interleukin-6 (IL-6), interleukin-1*β* (IL-1*β*), and calcidiol [25-(OH)D] levels were measured in blood serum using ELISA immunoenzymatic test kit (EIA-4640, EIA-4437, EIA-5396, DRG Instruments GmbH, Germany).

### 2.5. Statistical Analysis

The results were presented as arithmetic means and standard deviations (±SD). Conformity with normal distribution was checked with the Shapiro-Wilk test. The training-induced changes were checked with Student's *t*-test for dependent variables or the Mann–Whitney *U* test. The correlations between the studied variables were assessed using Pearson's correlation coefficient for variables with normal distribution. Spearman's rank correlation test was used for variables that did not comply with normal distribution. The level of significance was set at *p* < 0.05. STATISTICA 10.0 software (StatSoft Inc., Tulsa, OK, U.S.A.) was used for all statistical calculations.

## 3. Results

Tables 1–4 show the results of the study.  Table 1 presents the basic anthropometric characteristics of participants and statistical analysis results. The 12-week Nordic walking training program did cause a significant reduction in participants' body mass and fat mass (Δ = −2.5 ± 1.7, *p* = 0.0001; Δ = −3.8 ± 1.7, *p* = 0.03, resp.) and a significant increase in lean body mass (Δ = 1.3 ± 0.5, *p* = 0.04).  Table 2 shows the mean total distance covered by the women in particular training mesocycles.

Table 3 shows changes in the concentration of selected biochemical markers. The Nordic walking training program contributed to a significant increase in the TAS and calcidiol levels (*p* = 0.007 and *p* = 0.008, resp.).

The measurement before the training program revealed a reverse correlation between IL-6 concentration and TOC (*r* = −0.70, *p* = 0.046), and the measurement after the training program revealed a reverse correlation between IL-6 level and calcidiol (*r* = −0.91, *p* = 0.001). The correlations of selected results are shown in Table 4.

## 4. Discussion

In the present study, Nordic walking training, conducted three times a week for 12 weeks, was sufficient to increase physical performance, measured by the increment of covered distance in equal time (Table 2), and brought about a reduction in participants' body mass, fat mass and mean BMI and a simultaneous increase in lean body mass (LBM) (Table 1). The last increase is most probably an effect of applied resistive exercises as parts of the Nordic walking training. According to Ryan et al. [20], the use of such exercises in women exerts a positive influence on muscle endurance. They noted that a 16-week resistance training program in women (60 min, 3 times a week) applied to different muscle groups (arms, legs, chest, spine, and abdomen) resulted in an increase in muscle strength of the arms by 36–65% and that of the legs by 32–98%, despite a reduction in body mass and BMI (*p* < 0.05). Moreover, Ogawa et al. [21] found that the 12-week, low-intensity, resistance exercise training program caused an increase of muscle mass in elderly women.

In our study, the positive effects of Nordic walking were also confirmed by the lower oxidative stress levels and higher enzymatic and nonenzymatic antioxidative capacity in the blood of middle-aged individuals. The present study revealed a significant increase in the TAS levels (Table 3), which is in accordance with the results of our previous study [22] and Takahashi et al. [23]. A similar rise in TAS, following a six-week aerobic dancing training of moderate intensity in women aged 30–55 years, was noted by Leelarungrayub et al. [24]. The increased TAS levels observed in the present study with no concurrent changes in the TOC levels (Table 3) can be explained by the notion of hormesis proposed by Masoro [25], which assumes that even insignificant oxidative stress (associated with systematic physical activity) causes adaptative changes in different cell types and increases their antioxidative potential. Regular physical exercise leads to a rise in endogenous antioxidants levels, which are able to neutralize free radicals [10]. As a result, the cells become less susceptible to damage associated with acute oxidative stress. It appears then that even a small, chronic oxidative stress associated with training inducing a repeated increase in ROS improves the body's antioxidative defense, as noted by Finkel and Holbrook [26].

IL-1*β*, IL-6, and TNF-*α* are cytokines released most quickly in response to exercise stimuli [27]. The molecular mechanisms of the restoration of damaged muscle involve increases in IL-1, IL-6, and IL-10 [28]. IL-1*β* stimulates the synthesis of brain-derived neurotrophic factor (BDNF) which regulates the proliferation, differentiation, and survival of neurons and enhances the production of ROS taking part in gene expression [29]. IL-6 is a multidirectional cytokine which activates T lymphocytes, regulates the growth and diversity of B lymphocytes, and stimulates the hepatic secretion of molecular chaperones HSP (*heat shock proteins*), whose expression increases when the cells are exposed to stress factors, for example, high temperature or ROS [30]. IL-6 takes part in energy processes through regulation of glucose and FFA blood levels. IL-6 also stimulates lipolysis in the adipose tissue, elevates the blood levels of triglycerides and FFA, and enhances the *β*-oxidation of fatty acids in muscle cells [31]. The present study did not reveal changes in the levels of the examined cytokines; however, their role in the coordination of inflammatory and regenerative processes during muscle restoration, as indicated by Philippou et al. [32], should not be excluded. This is confirmed by a negative correlation between TOC and IL-6 in the initial measurement. Zembroń-Łacny et al. [33] found a high positive correlation between ROS markers and IL-6, which not only points to the role of ROS in the secretion of IL-6 regulation but also to its antioxidant modulation. On the other hand, Ogawa et al. [21] showed that low-intensity exercise training reduced levels of inflammatory markers and cytokines. These changes and also the inverse correlation between percentage changes in subscapular muscle thickness and percentage changes in CRP and TNF*α* suggest that low-intensity resistance training is beneficial for preventing sarcopenia in aged sedentary women.

The fact that Nordic walking does not induce inflammation in working muscles is indicated by a reverse correlation between IL-6 and 25(OH)D after the completion of the training program. Other authors showed that vitamin D deficiency contributed to the losses of muscle mass and strength [34, 35], which is related to a reduction of vitamin D receptors (VDR) in muscles [36]. Vitamin D has also anti-inflammatory properties, and its deficiency is associated with more severe inflammation. Schleithoff et al. [37] found that vitamin D_3_ supplementation contributed to a decreased secretion of proinflammatory factors such as CRP, TNF-*α*, and IL-6.

After finishing the 12-week Nordic walking training program, the increase in blood 25(OH)D level (up to 27.2 ± 4.9 ng·ml^−1^) was observed. The opposite phenomenon was observed in our previous study evaluating the effect of a 6-week long training conducted in the autumn-winter period (October–December) [38]. A decrease in vitamin D level was also observed after 32-week Nordic walking training in the study of Kortas et al. [39]. Despite the increased numbers of hours of sunshine during the months when the training was now conducted and maintaining current eating habits, the ultimate calcidiol level was still below the recommended standard values and it appears that external supplementation of this vitamin is needed.

In the planning of endurance efforts for middle-aged people, it is useful to introduce heart rate monitors, which in our group helped to enforce the intensity of march in the main part in the first mesocycle 50 ± 1.2% HRmax (average distance covered during classes 5.5 ± 0.2 km), in the second 60 ± 2.3% HRmax (average distance covered during the course 6.5 ± 0.3 km), and in the third 65 ± 3.1% HRmax (average distance covered during the course 7.8 ± 0.5 km), as it was shown in Table 2. An important role in the generation of biochemical and somatic changes in participants of the study was generated by the standardization of trainings understood by regularity of attending classes, individualization resulting from continuous HR measurement, instructor control of both the technique of performing movement as well as maintaining correct HR values, and increasing the marching intensity in subsequent mesocycles.

## 5. Conclusions

In conclusion, the results of the present study corroborate those concerning the impact of Nordic walking on body composition and some biochemical markers obtained in other studies. A 12-week Nordic walking training undertaken by premenopausal women not only had a positive effect on body composition but also on plasma antioxidative capacity. However, it cannot replace adequate dietary intake or substitution of vitamin D.

The use of heart rate monitors in each participant contributed to the individualization of conducted health training. Thanks to monitoring the heart rate during classes, instructors could precisely adjust the speed of march to the planned heart rate, all the while taking care of the proper technique of Nordic walking.

## Figures and Tables

**Table 1:** Selected somatic indices in the studied women.

	Baseline	After 12 weeks	∆	*p*
BM (kg)	63.8 ± 7.2	61.3 ± 5.8^∗^	−2.5 ± 1.7	0.0001
LBM (kg)	43.7 ± 3.2	45.0 ± 3.1^∗^	+1.3 ± 0.5	0.04
FM (kg)	20.1 ± 3.1	16.3 ± 2.9^∗^	−3.8 ± 1.7	0.03
BMI (kg·m^−2^)	23.4 ± 2.5	22.3 ± 1.8^∗^	−1.1 ± 0.5	0.02

BH: body height; BM: body mass; LBM: lean body mass; FM: fat mass; BMI: body mass index. ^∗^*p* < 0.05.

**Table 2:** Training volume in particular training mesocycles.

	Mesocycle 1	Mesocycle 2	Mesocycle 3
Covered distance (km)	5.5 ± 0.2	6.5 ± 0.3	7.8 ± 0.5

**Table 3:** Levels of selected biochemical markers in the studied women.

	Baseline	After training	∆	*p*
IL-1*β* (pg·ml^−1^)	2.6 ± 1.3	2.0 ± 0.6	−0.6 ± 1.1	0.13
IL-6 (pg·ml^−1^)	48.0 ± 15.1	44.0 ± 11.0	−4.0 ± 13.6	0.37
TOC (*μ*mol·l^−1^)	0.08 ± 0.03	0.09 ± 0.05	+0.01 ± 0.05	0.67
TAS (*μ*mol·l^−1^)	1.1 ± 0.4	1.6 ± 0.2^∗^	+0.5 ± 0.4	0.007
25(OH)D_3_ (ng·ml^−1^)	23.8 ± 5.7	27.2 ± 4.9^∗^	+3.5 ± 2.6	0.008

IL-1*β*: interleukin-1*β*; IL-6: interleukin-6; TOC: total oxidative capacity; TAS: total antioxidative status. ^∗^*p* < 0.05.

**Table 4:** Correlations between selected biochemical and somatic parameters.

	*r*	*p*
∆ 25(OH)D_3_ versus BM baseline	0.75	0.008
∆ 25(OH)D_3_ versus BM after training	0.80	0.003
∆ 25(OH)D_3_ versus ∆ BM	0.50	0.120
Il-6 baseline versus TOC baseline	−0.70	0.046
Il-6 after training versus 25(OH)D_3_ after training	−0.91	0.001

∆: difference between baseline and after training measurements, *r*: Pearson correlation coefficient. *n* = 13.
